# Field Study on Sow Mortality in 15 Belgian Pig Farms

**DOI:** 10.3390/vetsci12070603

**Published:** 2025-06-20

**Authors:** Caroline Bonckaert, Charlotte Brossé, Tamara Vandersmissen, Nermin Caliskan, Ellen Buys, Ilias Chantziaras, Dominiek Maes

**Affiliations:** 1 Animal Health Care Flanders, Hagenbroeksesteenweg 167, 2500 Lier, Belgium; 2 Unit of Porcine Health Management, Department of Internal Medicine, Reproduction and Population Medicine, Faculty of Veterinary Medicine, Ghent University, Salisburylaan 133, 9820 Merelbeke, Belgiumdominiek.maes@ugent.be (D.M.)

**Keywords:** sow mortality, genetics, nutrition, management, necropsy, pig farming

## Abstract

Sow mortality is a critical issue in intensive pig farming, impacting animal welfare, farm sustainability, and profitability. This study investigated the occurrence and causes of sow mortality on 15 Flemish sow farms, focusing on management practices, housing conditions, feeding strategies, and genetics. The average sow mortality rate across the farms was 11.4% in 2022, which decreased to 8.1% in 2023 following the implementation of targeted control measures. Necropsies performed on 100 deceased sows, coming from the 15 different farms, revealed that the primary causes of mortality were positional changes in internal organs (32%), arthritis (19%), and urogenital disorders (7%). Key recommendations to reduce sow mortality included optimizing sow health and body condition, improving housing and feeding management, and addressing genetic factors. The study highlights the multifaceted nature of sow mortality and the importance of a comprehensive approach to mitigate risks and improve sow welfare and productivity.

## Simple Summary

This study addresses the critical issue of sow mortality in intensive pig farming, which affects animal welfare, farm sustainability, and profitability. The research aimed to investigate the causes and occurrence of sow mortality on 15 Flemish pig farms, focusing on management practices, housing conditions, feeding strategies, and genetics. Over the course of the study, the average sow mortality rate decreased from 11.4% in 2022 to 8.1% in 2023 following the implementation of targeted control measures. Necropsies revealed that the primary causes of mortality were positional changes in internal organs, arthritis, and urogenital disorders. Key recommendations to reduce sow mortality included optimizing sow health and body condition, improving housing and feeding management, and addressing genetic factors. The study highlights the multifaceted nature of sow mortality and the importance of a comprehensive approach to mitigate risks and improve sow welfare and productivity. These findings are valuable to society as they provide actionable insights for farmers to enhance animal welfare and farm efficiency, ultimately contributing to more sustainable and profitable pig farming practices.

## 1. Introduction

Sow mortality has emerged as a significant concern, particularly in intensive farming systems. Recent studies in Flanders [1] report an average sow replacement rate of 40–45%, attributed to planned and unplanned culling. Planned culling, though often subjective, is typically based on factors such as lameness, high parity, or reduced productivity [2,3]. Conversely, unplanned culling is frequently associated with sudden death or other fast-evolving (<1 month) health issues [2,4]. These problems are often exacerbated by the increasing genetic selection for traits like prolificacy, which can inadvertently reduce the robustness and longevity of the sows [4]. 

Historically, data from 14 Flemish pig farms indicated a yearly sow mortality rate of 3.6% during 1995–2001 [5]. Also, according to The Danish Veterinary and Food Administration, sow mortality has been increasing steadily. In 2003, the annual sow mortality rate was approximately 12% [3]. Alarmingly, more recent observations (2018–2019) suggest that the mortality rate in many modern sow farms often surpasses 10%, with Danish studies reporting an average annual mortality rate of 14% (ranging from 6.4% to 18.5%) [6]. This upward trend in sow mortality is worrying, as it affects the profitability of pig farms and animal welfare. High mortality rates may indicate underlying systemic problems within the herd’s health and management protocols, necessitating a thorough examination of the contributing factors.

Sow mortality is a multifaceted problem that negatively impacts animal welfare, farm sustainability [7], and profitability [8]. The intensification of pig production, characterized by increased herd sizes and a focus on maximizing output, has placed additional pressure on the sows. The transition towards leaner genetics, driven by market demands, has inadvertently resulted in lower body fat reserves, making sows more susceptible to metabolic and reproductive disorders and heat stress. Moreover, the quality of stockmanship has not always kept pace with these changes [9]. 

Environmental factors, such as heat stress, have also been implicated as significant contributors to sow mortality [10,11]. Heat stress can lead to a cascade of physiological responses, including reduced feed intake, impaired thermoregulation, and increased disease susceptibility. The situation will likely worsen due to ongoing climate change, highlighting the need for management strategies to mitigate these risks [12]. Furthermore, inadequate housing and nutrition can exacerbate the problems, as insufficient space and suboptimal-quality diets contribute to health problems. The practice of feeding sows once daily, for example, has been associated with a higher risk of gastric torsions compared to more frequent feeding regimens [13]. 

Necropsies have proven to be a crucial diagnostic tool in identifying the underlying causes of sow mortality, particularly when clinical signs on herd level are absent or nonspecific [4,14,15]. Also, in Flanders, common necropsy findings in dead sows include torsions of internal organs (e.g., stomach, liver, spleen, intestines), heart failure, and gastric ulcers [16].

This study investigated the occurrence and causes of sow mortality on 15 Flemish sow farms. To this end, specific farm management practices, including housing conditions, feeding strategies, and herd health protocols were investigated, and necropsies were performed on a subsample of dead sows.

## 2. Material and Methods

### 2.1. Study Population

The study was announced via the Animal Health Care Flanders (DGZ) newsletter, which is sent to most pig farms in Belgium. Interested farms complying with the inclusion criteria, namely having at least 150 sows and an annual sow mortality rate of at least 5% in 2022, could participate. The 5% threshold was used as a selection criterion based on historical Belgian field data [5] and the reference provided in Diseases of Swine. Fifteen Flemish pig farms participated in the study.

### 2.2. Farm Visits

Each participating farm was visited three times over a period of 18 months. The first visits started in October 2022, and the final visits were completed by January 2024. The initial visit consisted of an inventory of relevant farm data through a questionnaire, along with an inspection of the farm facilities. During the second visit, approximately three months later, recommendations were provided regarding control measures aimed at reducing sow mortality. A third and final visit, conducted around six months after the second, served to evaluate any changes in sow mortality rates. The visits were carried out by the first and last authors, both of whom have training and experience in the assessment of pig welfare indicators. To ensure consistency and inter-observer reliability, several of the first visits were conducted jointly. When conducted separately, close coordination took place before and after each visit. As the study progressed, the last author conducted most of the second visits, while the final visits were either carried out together or individually, always followed by joint discussion of the observations to ensure alignment in scoring and interpretation.

### 2.3. Survey Design and Data Collection

The questionnaire consisted of several sections. The first section focused on general farm information (Table 1). The next four sections were related to specific farm units, namely the breeding gilt unit (Table 2), the insemination and gestation units (Table 3), and farrowing unit (Table 4).

### 2.4. Feed Composition and Analysis of the Drinking Water

Different aspects related to feed management of gilts, pregnant and lactating sows were recorded such as the feed composition, the feeding level, and the physical form of the feed (Table 3 and Table 4). Farmers could have the sow feed analyzed (Weende analysis) in the laboratory of Animal Nutrition of the Faculty of Veterinary Medicine at Ghent University, and the bacterial and chemical sow drinking water quality in the laboratory of DGZ Flanders. Weende analysis is a method used to determine the basic components of animal feed or food. It involves analyzing the moisture, ash, crude protein, crude fat, and crude fiber content of a sample. Bacterial analysis of the drinking water included number of Coliforms (cfu/100 mL), number of E. coli (cfu/mL), number of intestinal Enterococcus species (cfu/100 mL), number of sulfide-reducing Clostridia (cfu/20 mL), and total number of aerobe bacteria at 22 °C and at 37 °C (cfu/mL). Ammonia (mg/L), sodium (mg/L), nitrate (mg/L), nitrite (mg/L), and sulfide (mg/L) were analyzed in the chemical drinking water analysis, as well as the pH, the water hardness (°D), and salinity (mg/L).

### 2.5. Necropsy of Dead Sows

Throughout the study, each participating farm had the opportunity to present a maximum of eight deceased or euthanized sows for necropsy. Dead sows were picked up, and necropsies were performed in the necropsy room of DGZ. The aim was to have necropsied the sows within 24 h following notification of death. If the initial necropsy did not yield a conclusive diagnosis, additional diagnostic follow-up, e.g., PCR testing or microbiological culture followed by MALDI-TOF on specific matrices (tissues, body fluids) was performed to establish a conclusive diagnosis. The results were discussed with the farmers and the herd veterinarian during the next farm visit or, if needed, at an earlier stage.

### 2.6. Data on Deceased Sows

Farmers were asked to record specific information, such as parity and date of death of each sow that died during the study period (October 2022–January 2024).

### 2.7. Statistical Analysis

Excel was used to summarize and analyze the data descriptively. The number of farms per category was calculated for categorical variables; the mean, median, minimum, and maximum values for the continuous variables. The data were grouped according to the questionnaire structure, namely general farm characteristics, data from the breeding gilts, and the sows in the insemination, gestation, and farrowing unit.

The sow mortality results were calculated for the year before the study and the year of the implementation of the control measures, e.g., 2022 and 2023.

The necropsy results were shown descriptively for the different types of lesions, and given the high prevalence of torsions, further differentiation was made for the sows with torsions.

The sow mortality rates in the farms in 2022 and 2023 (before and after the implementation of control measures, respectively) were compared using the Wilcoxon signed-rank test. The parity distribution of dead sows in 2022 versus 2023 was analyzed using chi-square test. Sows with parity 7 or more were merged into one group to have sufficient numbers in each parity group. Two-sided tests were used. Differences were declared as statistically significant if *p* ≤ 0.05. All statistical analyses were performed using IBM SPSS version 29^®^ (Armonk, NY, USA).

## 3. Results

### 3.1. General Information of the Farms

The median number of sows per farm was 350 (min 180; max 950). Danish genetics were used in 11 of the 15 farms (73%) (Table 1). The median weaning age was 24.5 days (min 19.0; max 28.0 days), and the median number of pigs weaned per sow per year was 31.9 (min 26.1; max 36.9). The median annual sow replacement rate was 51.6% (min 29.3%; max 75%), over 14 herds as 1 herd had a replacement of 128%, as its capacity increased. A visual body condition score was performed on 60% (9 out of 15) of the farms, typically at 3–4 weeks of gestation or at weaning. Only four farms measured sow backfat thickness. Depending on the farm, these measurements were performed before farrowing, at weaning, or at 3–4 weeks of gestation.

### 3.2. Data of the Breeding Gilts

In 67% (10 out of 15) of the participating farms, breeding gilts were purchased (Table 2). The median number of purchases was 6.5 times per year (min 2.0; max 8.7). The purchased gilts stayed in a quarantine unit for 40.8 days (min 28.0 days; max 90.0 days) and had an average space of 1.3 m^2^ (min 0.7 m^2^; max 2.3 m^2^) per animal. The average group size was 14 animals (min 4; max 50) per pen.

### 3.3. Data of the Sows in the Insemination and Gestation Unit

In 33% (5 out of 15) of the participating farms, health and welfare issues were reported to occur regularly for sows in the insemination unit (Table 3). These problems were primarily coughing in gilts and young sows, and lameness.

In 33% (5 out of 15) of the farms, sows were fed once a day in the insemination unit. In 67% (10 out of 15) of the farms, sows were flushed with sucrose after weaning (min 5; max 7 days). Additionally, seven of the ten farms that practiced flushing of weaned sows also provided extra feed supplements such as Lianol^®^ (Ardol BV, Susteren, The Netherlands), benzoic acid, butyric acid, vitamin E, selenium, or a source of vitamins and minerals.

In 67% (10 out of 15) of the farms, welfare issues, mainly lameness or other locomotion problems, were frequently observed in the gestation unit (Table 3). On 9 of 10 farms experiencing locomotion problems, the sows were permanently housed in groups, without bedding material. In the other farms, the sows were not always loosely housed and could be temporarily and shortly locked in the crates. Additionally, on 20% (3 out of 15) of the farms, extra vitamin E and monocalcium phosphate were provided during gestation. The sows were moved from the gestation to the farrowing unit 6 days before expected farrowing (min 3.0; max 14.0 days).

### 3.4. Data of the Sows in the Farrowing Unit

Forty-seven percent (7 out of 15) of the farms regularly experienced health problems in sows in the farrowing unit, such as flu-like symptoms (fever, dyspnea), loss of appetite, or sudden death during the last week of gestation (Table 4). In most cases, the sows died without any prior clinical signs.

Animal welfare issues, such as shoulder ulcers or claw injuries, were reported on 67% (10 out of 15) of the farms. Additionally, 20% (3 out of 15) of the farms provided feed supplements such as wheat bran, sugar, or a vitamin/mineral preparation in the peripartal period.

### 3.5. Sow Mortality Throughout the Study

In 2022, sow mortality was greater than 15% in 27% of the farms. The average sow mortality in the participating farms was 11.4% in 2022, which decreased to 8.1% in 2023 (*p* < 0.01). There was a decrease in sow mortality in 12 farms (mean −3.2%; min −1.0%; max −10.4%), a slight increase in two farms, and no change in one farm (Table 5). Based on the data collected on sow mortality in 2022 and 2023, fewer sows died during November–December and February–March. In 2022, there was a peak in mortality in August, while in 2023, the peak occurred in July.

**Figure 1:**
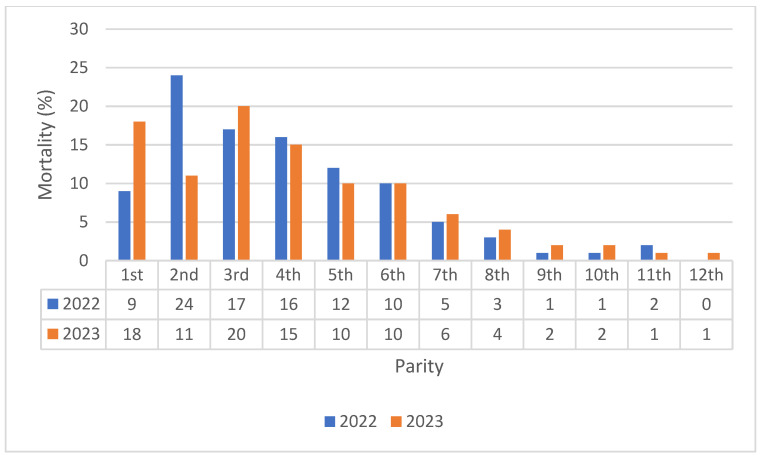
Sow mortality according to parity in the 15 farms in the year 2022 and 2023. The parity distribution was not statistically different (*p* > 0.05) between 2022 and 2023.

When looking at the parity of the deceased sows in both 2022 and 2023, mortality primarily occurred among young sows, with a peak during the second and third parity (Figure 1).

### 3.6. Necropsy Results of Dead Sows

**Figure 2:**
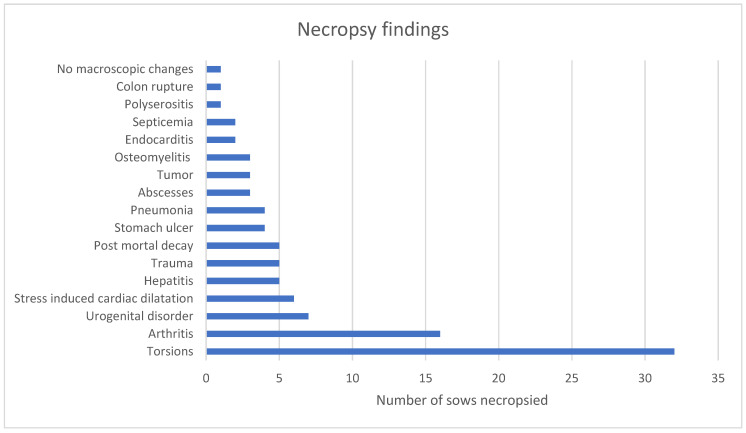
Results of the necropsy (y-as) of 100 sows (x-as). Main findings were torsions (n = 32), followed by arthritis (n = 16) and urogenital disorders (n = 7).

In total, 100 sows were necropsied (min 2; max 10 per herd). Most sows died due to positional changes in the intestines (Figure 2).

**Figure 3:**
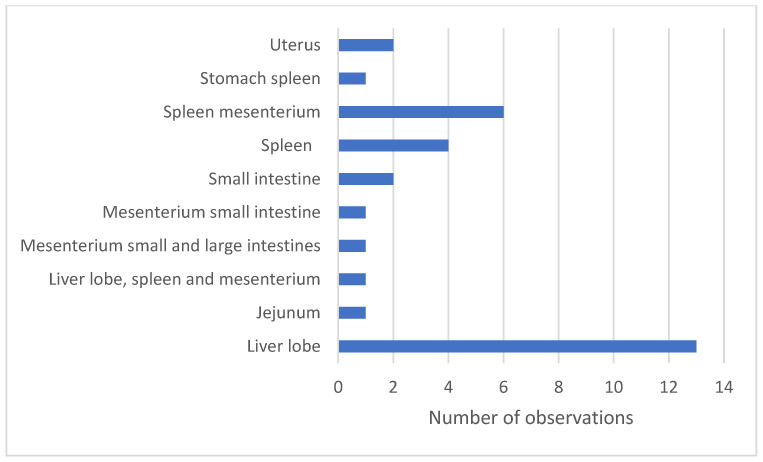
Number of sows with torsions in specific organs. Torsions were observed in 32% (n = 32) of the necropsied sows, originating from 14 farms.

Torsions of the liver lobes (41%), combined torsion of the spleen and mesenterium (19%), and spleen (13%) were the most frequently observed (Figure 3). The liver lobe torsions mainly involved the left lateral liver lobe.

**Figure 4:**
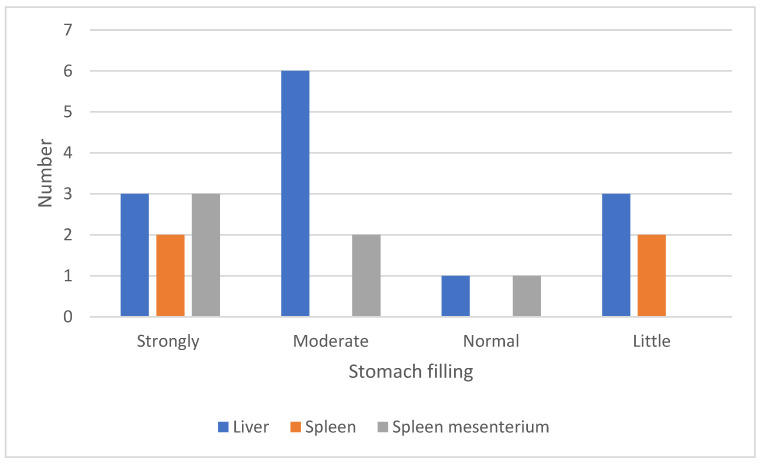
Link between the degree of filling of the stomach and finding of torsion of the liver (n = 13), spleen (n = 4), and spleen mesenterium torsions (n = 5). In one case of a spleen torsion the stomach filling was not specified and, therefore, taken out of the graph.

The stomach filling was checked when torsions were observed during necropsy (Figure 4). A strongly filled and a moderately filled stomach were observed in 33.3% and 37.5% of the cases with torsions, respectively. In 25% of the cases, the stomach filling was normal or low.

**Figure 5:**
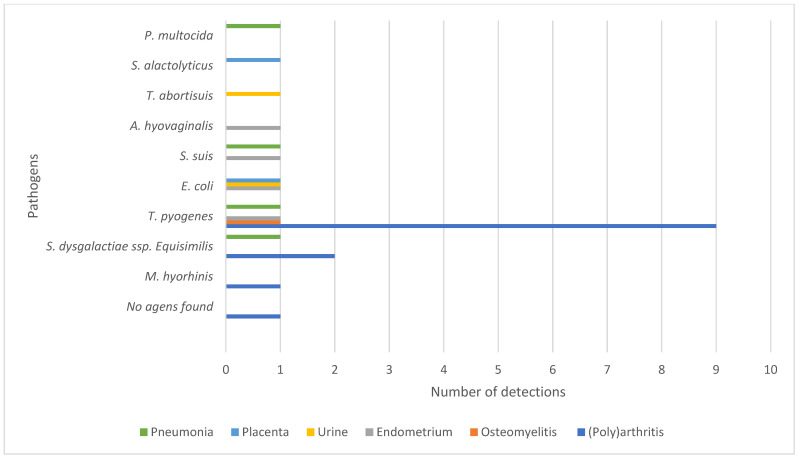
Pathogens found in lungs, placenta, urine, endometrium, or in lesions of osteomyelitis and (poly)arthritis.

Osteomyelitis and (poly)arthritis, both mostly purulent, were observed in 19 of the 100 necropsied sows *T. pyogenes* was most frequently isolated (53%) (Figure 5).

In six out of seven sows with urogenital disorders at necropsy, a culture of endometrium (n = 4), urine (n = 1), or placenta (n = 1) was conducted. In all cases, at least two bacteria were cultivated (Figure 5). Cystitis was diagnosed in one of the six sows. The urine was turbid, and varying amounts of pus or sediment was observed. The bladder mucosa appeared normal, but cortical cysts or small abscesses were frequently found.

### 3.7. Drinking Water Quality and Feed Composition

The water source was different between the farms: 9 farms used only deep well water, 2 used tap or public water, 1 surface water, 1 deep-drainage water, and 2 used a combination of deep well water and rainwater. The quality of the drinking water was analyzed in 6 out of 15 farmers. In the other farms, the drinking water quality had been tested before the study, and no major abnormalities were found. Therefore, no additional testing was performed during the study. From the six farms that analyzed the drinking water, the bacteriological water quality was good (n = 2), and in four farms, the concentrations of intestinal *Enterococci* (>1 cfu/100 mL) (n = 3) and/or *Clostridia* (>1 cfu/20 mL) (n = 2) were too high. In two farms, the hardness of the drinking water was too high (>20°D).

### 3.8. Main Recommendations

The general composition of the feed (Weende analysis) was analyzed on five farms. In some farms, only one type of feed was analyzed, and in other farms, two or three different feed were analyzed. In total, 10 feed samples were analyzed: gestation feed (n = 6 samples), lactation feed (n = 3 samples), and breeding feed (n = 1 sample). The protein level varied from 11.8% to 13.5% in the gestation feed, from 15.1% to 15.3% in lactation feed, and it was 14.1% in the breeding feed. The fat level in the gestation feed was 3.7% (min 2.8%; max 4.3%), in lactation feed 4.2% (min. 3.8%; max 4.8%), and in the breeding feed 4.1%. Crude fiber varied from 7.5% to 10.4% in gestation feed, from 4.5% to 5.5% in lactation feed and was 5.2% in the breeding feed.

The recommendations for reducing sow mortality primarily focused on optimizing animal health, feed composition, feeding management, housing conditions, and genetics (Table 6).

To improve herd immunity and disease prevention strategies, the evaluation of farm-specific sow vaccination schemes is essential. On five farms, the implementation of new vaccination programs targeting circulating pathogens was recommended, e.g., swine influenza, Glässer disease or *Clostridium difficile*. Additionally, on one farm, strict adherence to good hygiene practices was advised to limit disease transmission and enhance overall herd health. Furthermore, on two farms, the supplementation of vitamin E in feed and/or water was recommended, as it may support immune function and enhance vaccination efficacy. These combined measures contribute to a comprehensive health strategy aimed at improving sow welfare and productivity.

Several housing improvements were also recommended to further optimize sow welfare and productivity. First, in four farms, improving flooring conditions by preventing excessive moisture and slipperiness was suggested to minimize the risk of limb injuries and wounds. Second, in three farms, better climate control in the farrowing unit was advised to maintain stable temperatures, thereby reducing stress for both sows and piglets. Third, on two farms, the modernization and adaptation of farrowing crates were recommended to accommodate the larger body size of modern sows, preventing pressure wounds and enhancing comfort. Finally, on one farm, optimizing stocking density management was suggested to reduce competition and stress.

On six farms, increasing the crude fiber content in the gestation feed was advised. This recommendation was made due to high levels of aggression and/or limited resting opportunities in the gestation unit. In addition to increasing crude fiber content, the installation of escape partitions, e.g., extra walls, where sows could escape from other aggressive sows and relax, on one farm and/or additional feeding troughs on one farm was recommended to mitigate aggression in the gestation unit.

Lastly, on three farms with suboptimal sow body condition, it was advised to perform backfat thickness measurements in addition to visual body condition assessment to improve monitoring accuracy.

## 4. Discussion

The study results showed that sow mortality is high in many Flemish pig farms. Different potential risk factors related to nutrition, management, housing, and genetics could be identified on the farms. Together with the necropsy findings of dead sows, they were used to provide recommendations to decrease the sow mortality on the farms.

The study results confirmed that sow mortality is a multifaced problem. In 2022, the sow mortality rate was 11.4% on the Flemish participating farms. This confirms our concerns regarding increasing sow mortality rates, which already increased from 3.1% to 4.8% during the period 1995–2001 [5]. Finland also reported a sow mortality rate of 8.7%, and in Denmark, the sow mortality rate rose from 12.4% in 2018 to 15.1% in 2020 [6,17].

For the herd profitability and animal welfare, sows should remain on the farm during the most productive parities, i.e., 2 to 5 [18]. Therefore, gilts and first parity sows need to be healthy, to have a slower growth without excessive fattening [19]. Calcium and phosphorus concentrations must also be sufficiently high to prevent locomotory problems due to bone demineralization [20]. Consequently, particular attention should be given to the composition of the feed of gilts. In our study, lameness was indicated by the farmers as a major health and welfare issue on the farms, which was confirmed at necropsy. Therefore, advices concerning feed composition (sufficient crude fiber and protein) and body condition (visual and measurements) were given to eight farmers, with good results in sow mortality during the study (reduction of 3.86%).

Nineteen (19%) of the necropsied sows had osteomyelitis and/or (poly)arthritis. Sows spend most of their time in the gestation unit where they are housed in groups [21]. The social behavior of pigs is complex and heavily dependent on interactions with humans and other pigs. Most farmers indicated that lameness arose due to competition and aggression among the sows, which prevented some sows from eating calmly [3,22]. As a result, these sows could not consume their daily feed portion and had a lower body condition score. Farmers also noticed that the sows unable to eat often played with the drinking nipples, which led to a slippery floor, causing sows to slip and injure themselves and affecting animal welfare [22]. On 7 farms, it was advised to focus on rest and peace in the gestation stable. This was performed by installing an extra feeder, or to increase the amount of crude fiber in the feed. On one farm it was also advised to place fences so groups can be more separated and also important to lower the animal density.

Infectious causes can also affect sow health and increase the risk of death. On ten different farms, it was advised to monitor the health of the sows more often, to revise the vaccination scheme, or to administer extra vaccinations to prevent swine flu, Glässer disease, or *C. difficile*.

On 11 of the 15 participating farms with a high sow mortality, Danish genetics were used. Three farmers changed the sow genetics, not only because of the high sow mortality but also to reduce the workload in the farrowing units. Very high numbers of liveborn piglets require extra care and management to guarantee sufficient colostrum intake by the piglets, to keep them alive and healthy, and to ensure sufficient weaning weight. The associated costs were not assessed in this study. Furthermore, the decision to change genetics was not made rapidly. The number of sows of the other sow genetics was gradually increased, depending on the replacement rate. This decision is complex and significant, both practically and financially, and is, therefore, not feasible for every farmer. Farms that gradually transitioned to an alternative sow genotype showed a reduction in sow mortality. Given that organ torsions were the most frequently observed pathology, and that most participating farms used Danish genetics, this finding may not be surprising. A genetic predisposition—particularly related to mesenteric torsions—has been reported in the literature [23]. However, this aspect was not specifically addressed in the current descriptive study, and further research is needed to investigate this association in more detail.

The main findings of the necropsies can be divided into two groups: positional changes in internal organs (32%) and arthritis (19%). These findings are in agreement with other studies in Denmark [24], Canada [10], and the USA [7]. Urogenital disorders (7%) were the third most observed findings during necropsy. Urogenital disorders have been associated with lameness episodes [13]. Lame sows lie down for a longer time, urinate less, and have more contact with the floor, predisposing for ascending infections of the urinary tract [11]. Interestingly, these three main findings are all be influenced by feed and feed management [13], which was confirmed in our study. Changes in feed management may influence the risk for torsions of internal organs [25]. Hungry sows can become aggressive and fight. During the fights (micro)trauma in joints and bones easily can occur and therefore, sows are less able to stand up and drink [13]. Together with the above mentioned animal welfare issue, it is, therefore, logical that most of the recommendations were related to feed and feed management.

Torsions in Danish farms are commonly found, studies found 24% [3] and 42% [6]. This suggests that, in addition to nutrition, the genetics of the sows play a crucial role in sow mortality. Studies also show a relationship between feeding strategy and genetics. For example, crosses with Landrace or Large White pigs generally eat less frequently but larger quantities compared to other breeds, when given feed ad lib [26]. According to Abiven et al. the risk of sow mortality decreases when sows are fed more than once or twice a day. Spreading the daily diet over different meals may lower the speed of feed intake, which ultimately decreases the risk of organ torsions and sudden death [13]. This supports the association found between torsions and stomach filling. It was noted that in cases of spleen mesenteric torsions and liver lobe torsions, the stomach was moderately to heavily filled. Consuming large amounts of feed and possible anatomical variations in stomach size and attachment bands, or stress, may contribute to torsions [6,13,21], but further investigation is needed.

The main recommendations to improve sow welfare and survival included optimizing sow health and body condition, and minimizing aggression and restlessness within groups. These latter two factors are closely linked, as restlessness may trigger aggressive interactions, and vice versa. While feed composition and feeding strategies play an important role, group housing management is equally critical in maintaining optimal body condition. According to Verdon et al., pigs of similar size are more likely to engage in fights than those with size or age differences, as smaller animals are less likely to win confrontations [27]. This suggests that mixing sows of different parities could reduce aggressive encounters. However, our observations contrast with this assumption: first-parity sows were frequently affected by locomotor disorders, which we associate with injuries sustained during fights for dominance. This highlights the need for more targeted strategies when managing group composition, particularly to protect younger and more vulnerable individuals against locomotor disorders and thus improving animal welfare and animal health.

During necropsy, lesions including abscesses, trauma, osteomyelitis, and polyserositis were identified in 12 sows, coming from 7 different farms. In six cases, specific recommendations were made to improve housing conditions in both the gestation and farrowing units. These findings suggest that outdated farrowing pens may no longer accommodate the increased body size of modern sows, contributing to trauma and skin lesions that can serve as portals of entry for pathogenic bacteria. Subsequent bacterial spread may result in joint infections or systemic conditions. Maintaining the physical condition of the sow is therefore critical. A combined approach involving the enlargement of farrowing crates—e.g., using U-brackets—and tailored feeding strategies based on individual sow condition can help provide adequate space, reduce mechanical stress, and lower the risk of pressure wounds and secondary infections. The study was conducted on only 15 sow farms. The farms were not selected randomly, as the sow mortality had to be at least 5% to be eligible. Therefore, the sow mortality results are likely higher than the average Flemish sow farm. The farms were monitored during approximately 6 months, and mortality data for 2 successive years. Therefore, additional studies are needed to assess more long-term effects.

The 100 sows that were necropsied is a substantial number and provided a realistic picture of the studied farms. However, for some farms, it was not reported whether sows had died naturally and were euthanized.

In conclusion, sow mortality was high and ranged from 5% to 22% between the farms. The high mortality in hyperprolific sows represents an important welfare problem and needs to be properly addressed by the pig industry. The overall reduction in mortality from 11.4% in 2022 to 8.1% in 2023 reflects an average decrease of 3.2%. However, this change varied considerably across the 15 farms, with most farms exhibiting a decline in mortality ranging from 1.0% to 10.4%, while a few farms showed slight increases of up to 2.5%. Positional changes in internal organs, arthritis, and urogenital disorders were the most common necropsy findings. The farm recommendations differed between farms and were related to sow management, housing, and nutrition. The effects on herd level are mainly due to the analysis of the causes and the implementation of recommendations.

## Figures and Tables

**Table 1:** Sow genetics and batch farrowing systems on the 15 sow farms participating in the study.

	Number of Farms
**Sow Genetics**	
PIC ^a^	1
TN70 ^a^	2
Hypor	2
Danbred	11
**Batch Farrowing System for Sows**	
1-week	1
2.5-week	1
3-week	4
4-week	6
5-week	3

^a^ One farm had a combination of PIC and TN70 sows.

**Table 2:** Results of categorical variables related to the breeding gilts in the 15 farms.

	Number
**Purchase Gilts**	
Yes	10
No	5
**Feed**	
Dry	14
Liquid	1
**Physical Form of the Dry Feed**	
Pellets	4
Meal	10
**Ad lib Feeding Upon Purchase**	
Yes	10
No	5

**Table 3:** Results of the categorical variables related to the insemination unit and gestation unit in the 15 farms.

	Insemination Unit Number of Farms	Gestation Unit Number of Farms
**Presence of Health Problems**		
Yes	5	5
No	10	10
**Presence of Welfare Problems**		
Yes	4	10
No	11	5
**Insemination Unit Together with Gestation Unit**		
Yes	2	
No	13	
**Housing System**		
Temporarily locked in the crates	15	6
Group	0	9
**Ventilation System**		
Natural	3	2
Mechanical valve	4	8
Mechanical combi	3	0
Mechanical channel	4	5
Mechanical roof	1	0
**Problems with Climate in the Stable**		
Yes	0	2
No	15	13
**Type of Feed**		
Lactation	1.5	0
Estrus stimulation	6.5	0
Gestation	7	15
**Number of Feeding Phases**		
1-phase	15	10
2-phase	0	5
**Physical Form**		
Meal	6	6
Crumble	0	1
Pellet	8	7
Liquid	1	1
**Ad Lib Drinking Water**		
Yes	13	14
No	2	1
**Water Supply**		
Nipple	10	10
Trough	4	0
Via liquid feed only	1	1
Combination of nipples and troughs	0	4
**Treatment of the Drinking Water**		
Yes	7	7
No	8	8
**Type of Drinking Water Treatment**		
Organic acids	0	0
Peroxide	5	5
Combination of organic acids and peroxide	2	2

**Table 4:** Results of the categorical variables related to the farrowing unit in the 15 farms.

	N
**Presence Health Problems**	
Yes	7
No	8
**Presence Welfare Problems**	
Yes	10
No	5
**Housing System**	
Conventional farrowing crates	14
Balanced-framed farrowing crates *	1
Free-farrowing pens	0
**Air Inlet of the Mechanical Ventilation System, via…**	
Valve	0
Roof	2
Door	1
Channel below the corridor	7
Tube near the head of the sow	5
**Abnormalities in Climate**	
Yes	4
No	11
**Feed Management**	
Gestation feed followed by lactation feed 1 phase	2
Gestation feed followed by lactation feed 2 phases	1
Immediately lactation feed 1 phase	3
Immediately lactation feed 2 phase	3
Transition feed followed by lactation feed 1 phase	6
**Physical Form of Feed**	
Meal	7
Crumble	0
Pellet	7
Liquid	1
**Ad Lib Drinking Water**	
Yes	15
No	0
**Type of Drinking Water Treatment**	
Organic acids	5
Peroxide	2
Combination of organic acids and peroxide	4
None	4

*: a balanced-framed farrowing crate is a type of farrowing pen designed to optimize sow welfare and piglet survival by providing adequate space and support for both the sow and her litter. A key feature is that when the sow is lying down, her floor is level with that of the piglets, allowing easy access for nursing. However, when the sow stands up to eat, the floor beneath her is raised higher than the piglets’ floor. This design helps reduce piglet crushing.

**Table 5:** Sow mortality in 15 farms before (2022) and after the study (2023).

	Mortality in 2022	Mortality in 2023	Difference (2023–2022)
1	17.8	16.2	−1.6
2 *	22.0	13.0	−9.0
3 *	13.7	5.9	−7.8
4	17.2	11.8	−5.4
5	12.5	10.6	−1.9
6	20.0	9.6	−10.4
7 *	6.0	3.6	−2.4
8 *	6.4	7.6	+1.2
9 *	10.6	6.3	−4.3
10	10.0	10.2	+0.2
11 *	5.0	3.4	−1.6
12 *	7.6	4.1	−3.6
13 *	5.9	4.8	−1.0
14 *	6.9	3.7	−3.2
15 *	8.8	11.3	+2.5
**Mean**	**11.4**	**8.1**	**−3.2 ^a^**
**Median**	**10.0**	**7.6**	**−2.4**
**Min**	**5.0**	**3.4**	**+2.5**
**Max**	**22.0**	**16.2**	**−10.4**

^a^: The difference between the sow mortality rate in 2022 versus 2023 was statistically significant (*p* < 0.010); * these farms optimized at least their health policy in order to reduce sow mortality.

**Table 6:** Main recommendations to decrease sow mortality on the 15 farms.

Observation	Number of Different Farms	Advices
***Suboptimal Health Policy***	10	**Adaptation vaccination schemes (n = 7)** ^a^
		**Control disease (n = 4)**
		**Supplementation (e.g., vit E** to 100 ppm**) (n = 3**)
		**Hygiene protocol during vaccination (n = 1)**
***Suboptimal Housing***	6	**Prevention slippery floors (n = 4)**
		**Adaptation temperature in stable (n = 2)** (e.g., not higher than 25 °C)
		**Broadening boxes** to wider farrowing crates **(n = 2)**
		**Adaptation climate (n = 1)** (prevention draught)
		**Lowering animal density (n = 1)**
***Suboptimal Genetic Conditions***	6	**Changing sow and/or boar genetics**
***Suboptimal Body Condition***	8	**Body condition determination (weight, measurements) (n = 3)**
		**Adaptation feed composition** (**amount of proteins**) (**n = 2**)
		**Include more feed stations (n = 1)**
		**More feeding times in the farrowing unit** (from 2 to 4 times feeding) (**n = 1**)
***Aggression and Restlessness***	7	**Installation of water flow meters (n = 1)**
		**More crude fiber in feed (n = 1)**
		**Prevention restlessness (n = 1**)/encouraging calmth and silence
		**Installation of extra feeders (n = 1)**
		**Provide more kg of feed (n = 1)**
		**Calmth at replacement of the sows (n = 1)** ^b^
		**Lowering fighting possibilities (n = 1)**
		**Changing feeder type (n = 1)**
***Suboptimal Water Management***	3	**Analysis of water quality (n = 2)** and optimization
		**Provide** extra **water in the farrowing unit (n = 1)**
		**Measurement water flow (n = 1)**
***Suboptimal Parity Distribution***	3	**Purchase more gilts (n = 2)**: replacement rate from 29.7% to 38.9%
		**Prevention shoulder injuries (n = 1)**
***Suboptimal leg quality***	3	**Supplementation feed additives (n = 2)**
		**Separation sow groups (n = 1)**
***Suboptimal Gilt Management***	1	**Inseminate gilts at a younger age (n = 1)**: from 275 days to 259 days
		**Ad lib feeding (n = 1)**
***Suboptimal Feed Management***	**1**	**More crude fiber in feed (n = 1)** (15% instead of 13.5%).

^a^: starting vaccination against swine influenza in sows and gilts (n = 2), against *C. difficile* (n = 2) in sows and gilts, against Glässer disease in gilts (n = 1); ^b^: e.g., moving sows quietly from gestation to farrowing.

## Data Availability

The data are not publicly available due to ethical restrictions regarding participant confidentiality.
